# Preview Control with Dynamic Constraints for Autonomous Vehicles

**DOI:** 10.3390/s21155155

**Published:** 2021-07-29

**Authors:** Rui Li, Qi Ouyang, Yue Cui, Yang Jin

**Affiliations:** School of Automation, Chongqing University, Chongqing 400044, China; 20153973@cqu.edu.cn (R.L.); cysekiro@foxmail.com (Y.C.); 20173967@cqu.edn.cn (Y.J.)

**Keywords:** autonomous vehicles, preview control, dynamic constraints

## Abstract

In this paper, a preview theory-based steering control approach considering vehicle dynamic constraints is presented. The constrained variables are predicted by an error states system and utilized to adjust the control law once the established dynamic constraints are violated. The simulated annealing optimization algorithm for preview length is conducted to improve the adaptability of the controller to varying velocities and road adhesion coefficients. The theoretical stability of a closed-loop system is guaranteed using Lyapunov theory, and further analysis of the system response in time domain and frequency domain is discussed. The results of simulations implemented on Carsim–Simulink demonstrate the favorable performance of the proposed control in tracking accuracy and system stability under extreme conditions.

## 1. Introduction

The demands of traffic safety as well as the advances in sensing technology arouse researchers’ attention on autonomous vehicles and facilitate the development of this field [1,2]. Vehicle motion control is a key technology in autonomous vehicles, as it has a direct effect on tracking performance and safety [3]. This paper is concerned with the path tracking control of autonomous vehicles, which aims at ensuring the vehicle to accurately follow a reference trajectory and maintain stability under varying environmental and vehicular conditions.

Previous research associated with path tracking can be roughly classified into three categories. (1) The first is geometric control. Pure pursuit [4,5], vector pursuit [6], and the Stanley algorithm [7,8] are three typical geometric-based methods in which the steering angle is computed by the geometric relationship between the kinematics model and target point. One of the primary benefits of this control is that it is simple to implement, but the vehicle may exhibit performance degradation and even instability as speeds are increased due to the neglect of vehicle dynamics. (2) The second category is dynamics-based feedback control without prediction, including state feedback control [9,10,11], sliding-mode control [12], and *H*_∞_ control [13,14]. Kritayakirana et al. [9] held the view that using the center of percussion as the control point decoupled the vehicle’s lateral motion and yaw motion, which simplified the solution of the steering angle. On the premise of this concept, they proposed a liner state feedback controller and verified the stability of the closed-loop system utilizing Lyapunov theory. Kapania et al. [10] incorporated the desired sideslip behavior into the feedforward term and designed a feedback–feedforward steering control algorithm to enhance the vehicle performance at the limits of handling. A sliding-mode controller was presented by Imine and Madani [12] to prevent the lane departure of heavy vehicles. Considering the impact of model uncertainties on driving stability, a *H*_∞_ control via scenario optimization was applied to stabilize the vehicle in [13]. Mammar et al. [14] proposed an active steering method using robust control theory for vehicle handling improvement. This controller utilized the feedback action of yaw rate and allowed for driver steering input by adjusting the feedfoward operation. The aforementioned methods of this category can usually achieve favorable tracking behaviors in normal cases, but they may fail to acquire satisfactory results under highly dynamic conditions mainly due to the absence of future road information and vehicle dynamic constraints. (3) The model predictive control (MPC) scheme employs a vehicle dynamic model to forecast the future evolution of the system and generate online open-loop optimal control input in a receding horizon under the consideration of constraints [15,16]. Due to the requirement of optimization at each sampling time, numerical computation is inevitable, which may trigger heavy burden on a vehicular computer. Borrelli et al. [17] proposed a nonlinear model predictive control (NMPC) algorithm to stabilize the vehicle while reaching the physical constraints. Only simulation was conducted because of high computational complexity. To satisfy the demands of real-time control, researchers pour more attention into linear time-varying model predictive control (LTV-MPC), which is the MPC with a linear model. Compared with NMPC, LTV-MPC obtains more economical computing solution at the loss of minor tracking accuracy [2]. Falcone et al. [18,19] presented an LTV-MPC based on successive online linearization of a nonlinear vehicle model. By constraints on the tire slip angle, this controller achieved acceptable performance at high speeds and alleviated the intensive computations as compared to NMPC. An improved LTV-MPC method involving estimations of steering angle and state variables within the prediction horizon was further put forward in [20]. More recently, He et al. [16] designed a two-layer controller for path tracking, in which the upper layer was an LTV-MPC with parameters optimized via particle swarm optimization, and the lower layer consisted of a self-adaptive PID controller. Simulation results showed the robustness of this hierarchical controller under varying velocities and road adhesion. Despite the enhancement of computation efficiency in LTV-MPC, the numerical optimization of MPC may bring fluctuating values and even fail due to improper initial values [21]. Meanwhile, the explicit stabilizing conditions of MPC still remain to be coped with.

To achieve the simplicity of explicit control law in feedback control and hold the advantage of prediction and constraints in MPC, this paper presents a preview control considering vehicle dynamic constraints, in which the constrained variables are predicted via an error states model. Differing from the numerical optimization of MPC, the preview control can lead to an analytical solution with the coming road information as a priori using linear quadratic optimal theory [22]. A few studies focusing on preview control of autonomous vehicles are found in [23,24,25,26]. Among these proposals, Xu et al. did the most comprehensive work involving implementation, analysis, and experiments verification of preview path tracking control at low and middle speeds [24]. However, none of individuals have attempted to combine the preview control with dynamic constraints to tackle the stability issues of a vehicle under extreme conditions. Moreover, there exists no general algorithm to determine the optimal preview length except for the trial-and-error method, incurring low efficiency of controller design [22].

The contributions of this paper are presented as follows. (1) First, there is a preview controller considering vehicle dynamic constraints. An augmented system is formed via the future road curvature incorporated into error states, in which the control law is obtained with an analytical form. Then, the constrained variables are forecast relying on a linear errors model, and we determine the correction of the control law in accordance with the established constraints. (2) Second, there is the optimization of preview length by utilizing the simulated annealing algorithm. This process takes distinguishing vehicle speeds and road friction coefficients into account and operates offline. (3) Third, we present an analysis of the closed-loop system theoretical stability, steady-state response, and frequency response.

The remainder of this paper is organized as follows. Section 2 demonstrates the vehicle lateral dynamics model. Section 3 presents the design of preview control with constraints and the optimal preview length. The closed-loop system is analyzed in Section 4. Section 5 describes the simulation results, and Section 6 concludes this paper.

## 2. Vehicle Lateral Dynamics

**Figure 1:**
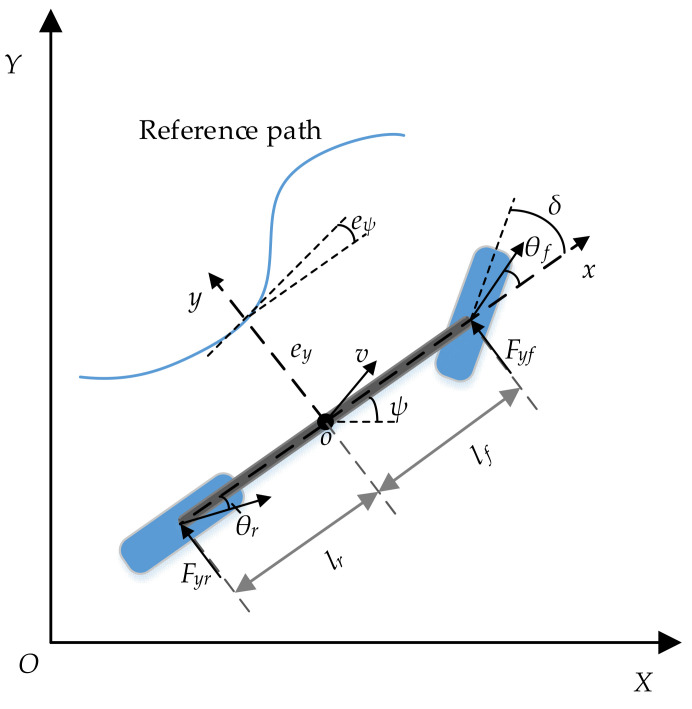
Vehicle dynamics model with a reference path.

Considering the trade-off between complexity and precision, a single-track dynamics model is adopted to develop the path tracking controller, as shown in Figure 1. The definitions are listed in Table 1.

Based on Newton’s second law, the force balance equation along the *y*-axis is

(1) $$m(\ddot{y}+\dot{\psi }v_{x})=F_{yf}+F_{yr}.$$

The moment balance equation in the yaw direction is

(2) $$I_{z}\ddot{\psi }=l_{f}F_{yf}-l_{r}F_{yr}.$$

We assume the tire slip angle is relatively small. Consequently, tire lateral force is proportional to slip angle [27]:

(3) $$F_{yf}=2C_{\alpha_{f}}\alpha_{f}=2C_{\alpha_{f}}(\delta -\theta_{f})$$

(4) $$F_{yr}=2C_{\alpha_{r}}\alpha_{r}=2C_{\alpha_{r}}(-\theta_{r})$$

where *θ_f_*/*θ_r_* is the velocity angle of the front/rear wheel. Under the assumption of small slip angle, they can be expressed as

(5) $$\theta_{f}=(\dot{y}+l_{f}\dot{\psi })/v_{x}$$

(6) $$\theta_{r}=(\dot{y}-l_{r}\dot{\psi })/v_{x}.$$

Substituting Equations (3)–(6) into (1) and (2), complete vehicle lateral dynamics are derived as follows:

(7) $$\ddot{y}=-\frac{2C_{\alpha f}+2C_{\alpha r}}{mv_{x}}\dot{y}-(v_{x}+\frac{2C_{\alpha f}l_{f}-2C_{\alpha r}l_{r}}{mv_{x}})\dot{\psi }+\frac{2C_{\alpha f}}{m}\delta$$

(8) $$\ddot{\psi }=-\frac{2C_{\alpha f}l_{f}-2C_{\alpha r}l_{r}}{I_{z}v_{x}}\dot{y}-\frac{2C_{\alpha f}l_{f}{}^{2}+2C_{\alpha r}l_{r}{}^{2}}{I_{z}v_{x}}\dot{\psi }+\frac{2C_{\alpha f}l_{f}}{I_{z}}\delta .$$

For the sake of concentrating on path tracking, dynamics models using error variables instead of vehicle variables are more effective. Consequently, we define the lateral error *e_y_* replacing *y*. The yaw angle error *e_ψ_* instead of *ψ* is defined as follows:

(9) $$e_{\psi }=\psi -\psi_{des}$$

where *ψ_des_* is the desired yaw angle, which is derived from the reference path. Considering that the vehicle runs along the lane with road curvature *ρ*, the desired yaw rate can be expressed as

(10) $${\dot{\psi }}_{des}=v_{x}\rho$$

where *v_x_* is the longitudinal velocity. The derivatives of *e_y_* and *e_ψ_* are defined as

(11) $$\left\{\begin{array}{c} {\dot{e}}_{y}=\dot{y}+v_{x}e_{\psi }\\ {\ddot{e}}_{y}=\ddot{y}+v_{x}{\dot{e}}_{\psi }\\ {\dot{e}}_{\psi }=\dot{\psi }-{\dot{\psi }}_{des}\\ {\ddot{e}}_{\psi }=\ddot{\psi }-{\ddot{\psi }}_{des} \end{array}\right..$$

Substituting Equations (9)–(11) into (7) and (8), transforming them into the state-space form, path tracking error dynamics are built as given in Equation (12):

(12) $$\left[\begin{array}{c} {\dot{e}}_{y}\\ {\ddot{e}}_{y}\\ {\dot{e}}_{\psi }\\ {\ddot{e}}_{\psi } \end{array}\right]=\left[\begin{array}{cccc} 0 & 1 & 0 & 0\\ 0 & -\sigma_{1}/v_{x} & \sigma_{1} & -\sigma_{2}/v_{x}\\ 0 & 0 & 0 & 1\\ 0 & -\sigma_{3}/v_{x} & \sigma_{3} & -\sigma_{4}/v_{x} \end{array}\right]\left[\begin{array}{c} e_{y}\\ {\dot{e}}_{y}\\ e_{\psi }\\ {\dot{e}}_{\psi } \end{array}\right]+\left[\begin{array}{c} 0\\ 2C_{\alpha f}/m\\ 0\\ 2C_{\alpha f}l_{f}/I_{z} \end{array}\right]\delta +\left[\begin{array}{c} 0\\ -v_{x}^{2}-\sigma_{2}\\ 0\\ -\sigma_{4} \end{array}\right]\rho$$

where *σ_i_* is defined as

(13) $$\begin{array}{l} \sigma_{1}=(2C_{\alpha f}+2C_{\alpha r})/m\\ \sigma_{2}=(2C_{\alpha f}l_{f}-2C_{\alpha r}l_{r})/m\\ \sigma_{3}=(2C_{\alpha f}l_{f}-2C_{\alpha r}l_{r})/I_{z}\\ \sigma_{4}=(2C_{\alpha f}l_{f}{}^{2}+2C_{\alpha r}l_{r}{}^{2})/I_{z} \end{array}.$$

As shown in Equation (12), the error state vector is [$e_{y}$,${\dot{e}}_{y}$,$e_{\psi }$,${\dot{e}}_{\psi }$]*^T^*, control input is the front wheel steering angle *δ*, and the road curvature *ρ* is treated as disturbance. The error state vector [$e_{y}$,${\dot{e}}_{y}$,$e_{\psi }$,${\dot{e}}_{\psi }$]*^T^* can be denoted by *x*; subsequently, Equation (12) is simplified as

(14) $$\dot{x}=Ax+B\delta +D\rho .$$

## 3. Controller Design

### 3.1. Preview Controller

The existence of disturbance road curvature *ρ* brings about the nonlinearity in Equation (14). Classical linear control methods, such as the linear quadratic regulator (LQR), are inadequate to handle this system. If the disturbance *ρ* is zero, the system becomes a linear one, in which LQR can be invoked to deal with. However, the designed controller not considering the disturbance *ρ* can only respond to this disturbance passively, resulting in poor performance, especially when driving on a fast-changing crooked road. Model predictive control (MPC) can solve this problem of nonlinear characteristics by online numerical optimization. Nevertheless, it may cause heavy computational burden considering the time-varying disturbance *ρ* and has difficulty in generating real-time control commands. Preview control is another suitable method with analytical solution. The future disturbance is incorporated into an augmented state vector to formulate an augmented LQR problem [22]. Therefore, we can obtain an analytical optimal solution based on optimal control theory.

In order to facilitate controller design and implementation, the continuous vehicle dynamics model in Equation (14) is converted into a discrete one with a fixed sampling period *T*:

(15) $$x(k+1)=A_{k}x(k)+B_{k}\delta (k)+D_{k}\rho (k)$$

where

(16) $$A_{k}=I+AT,\;B_{k}=BT,\;D_{k}=DT.$$

Then, we consider the finite previewed information of road curvature *ρ* in [*k*, *k*+*H*], where *H* is the preview length, which is available for control. This information can be denoted as

(17) $$x_{\rho }(k+1)=A_{\rho }x_{\rho }(k)=\left[\begin{array}{cccc} 0 & 1 & & 0\\ & 0 & \ddots & \\ & & \ddots & 1\\ 0 & & & 0 \end{array}\right]x_{\rho }(k)$$

where

(18) $$x_{\rho }(k)={\left[\rho (k),\rho (k+1),\cdots ,\rho (k+H)\right]}^{T}.$$

Subsequently, the augmented state vector is represented as

(19) $$\tilde{x}(k)=\left[\begin{array}{c} x(k)\\ x_{\rho }(k) \end{array}\right].$$

The augmented system becomes

(20) $$\tilde{x}(k+1)={\tilde{A}}_{k}\tilde{x}(k)+{\tilde{B}}_{k}\delta (k)$$

where

(21) $${\tilde{A}}_{k}=\left[\begin{array}{cc} A_{k} & {\tilde{D}}_{k}\\ 0_{(H+1)\times 4} & A_{\rho } \end{array}\right],{\tilde{B}}_{k}=\left[\begin{array}{c} B_{k}\\ 0_{(H+1)\times 1} \end{array}\right],{\tilde{D}}_{k}=\left[D_{k},0_{4\times H}\right].$$

For the augmented plant in Equation (20), standard LQR theory can be invoked to achieve an analytical solution satisfying the linear quadratic performance index:

(22) $$J=\sum_{k=0}^{\infty }[{\tilde{x}}^{T}(k)Q\tilde{x}(k)+R\delta^{2}(k)]$$

where *Q* is a positive semi-definite matrix and *R* > 0.

Minimizing *J* yields the optimal control input $\delta^{*}(k)$:

(23) $$\delta^{*}(k)=-K\tilde{x}(k)=-{\left(R+{\tilde{B}}_{k}{}^{T}P{\tilde{B}}_{k}\right)}^{-1}{\tilde{B}}_{k}{}^{T}P{\tilde{A}}_{k}\tilde{x}(k)$$

where *K* is the feedback gain, and *P* satisfies the difference Riccati equation:

(24) $$P={\tilde{A}}_{k}{}^{T}P{\tilde{A}}_{k}-{\tilde{A}}_{k}{}^{T}P{\tilde{B}}_{k}{\left(R+{\tilde{B}}_{k}{}^{T}P{\tilde{B}}_{k}\right)}^{-1}{\tilde{B}}_{k}{}^{T}P{\tilde{A}}_{k}+Q.$$

In order to better understand the optimal control, further decoupling $\tilde{x}(k)$ in Equation (23) into x(k),$x_{\rho }(k)$, the control input is divided into two parts as follows:

(25) $$\delta^{*}(k)=-K_{x}x(k)-K_{\rho }x_{\rho }(k)$$

where *K_x_* is the feedback gain vector corresponding to the error state *x*(*k*), and *K_ρ_* is the feedforward gain vector associated with the future road curvature. These two parts play different roles in control action: the first part called feedback aims to eliminate the deviations of path tracking; the second feedforward part deals with the disturbance resulting from road curvature. This term enhances steering adaptation to bend, thus improving the tracking accuracy.

### 3.2. Establishment of Vehicle Dynamic Constraints

It should be noticed that the optimal control in Equation (23) is derived without considering vehicle dynamic constraints; thus, this control is unfortunately inapplicable at the limits of the maneuvering capability of the vehicle. In addition, control with constraints is a difficult problem in optimal control theory [22,28]. To deal with this challenge, we proposed a sub-optimal control with constraints. By reasonably adjusting the optimal control feedback gain *K* in Equation (23), the driving stability is improved significantly.

Three aspects of constraints are considered as follows:

1. Mass center slip angle. Considering that the too large mass center slip angle *β* has a potential effect on the driving stability, the limited range of *β* is denoted as
  (26) −arctan(0.02μg)≤β≤arctan(0.02μg)
  where *μ* is the friction coefficient of the road, and *g* is the acceleration of gravity.
2. Tire slip angle. Due to the fact that the vehicle dynamics model is established under the small slip angle assumption, the tire slip angle beyond the linear region may incur lower model precision. Moreover, once the tire adhesion reaches saturation (namely beyond linear region), the vehicle may easily skid. For the sake of the aforementioned reasons, the following constraints are set to avoid vehicle instability:
  (27) $$\alpha_{\min}\leq \alpha \leq \alpha_{\max}$$
  where *α* represents both front tire slip angle *α_f_* and rear tire slip angle *α_r_*.
3. Input steering angle. This is a hard constraint based on vehicle physical limits and driving safety requirement that is imposed to ensure a reasonable range of steering angle:
  (28) $$\delta_{\min}\leq \delta \leq \delta_{\max}.$$

To implement the constraints mentioned above, the dynamics model in Equation (15) is adopted to predict the vehicle state during the previewed interval [*k*, *k* + *H*]. Note that the mass center slip angle and tire slip angle cannot be obtained directly in the state vector of Equation (15); thus, an observer is designed in the following.

The mass center slip angle *β* is denoted as

(29) $$\beta =\frac{\dot{y}}{v_{x}}=\frac{1}{v_{x}}{\dot{e}}_{y}-e_{\psi }.$$

Based on Equations (3)–(6), the tire slip angle α*_f_* and α*_r_* can be represented as

(30) $$\alpha_{f}=-\frac{1}{v_{x}}{\dot{e}}_{y}+e_{\psi }-\frac{l_{f}}{v_{x}}{\dot{e}}_{\psi }+\delta -l_{f}\rho \;\alpha_{r}=-\frac{1}{v_{x}}{\dot{e}}_{y}+e_{\psi }+\frac{l_{r}}{v_{x}}{\dot{e}}_{\psi }+l_{r}\rho .$$

Subsequently, by discretizing Equations (29) and (30), incorporating them into an observation vector, the constrained variables observer is obtained as follows:

(31) $$y_{o}(k)=C_{ok}x(k)+B_{ok}\delta (k)+D_{ok}\rho (k)$$

where

(32) $$y_{o}(k)=\left[\begin{array}{c} \beta (k)\\ \alpha_{f}(k)\\ \alpha_{r}(k) \end{array}\right],C_{ok}=\left[\begin{array}{cccc} 0 & 1/v_{x}(k) & -1 & 0\\ 0 & -1/v_{x}(k) & 1 & -l_{f}/v_{x}(k)\\ 0 & -1/v_{x}(k) & 1 & l_{r}/v_{x}(k) \end{array}\right],B_{ok}=\left[\begin{array}{c} 0\\ 1\\ 0 \end{array}\right],\;D_{ok}=\left[\begin{array}{c} 0\\ -l_{f}\\ l_{r} \end{array}\right].$$

Equations (15) and (31) deliver the prediction of constrained variables within the preview window [*k*, *k* + *H*]. If any constraints (except for the hard constraint for steering angle) are violated during this preview window, the optimal feedback gain *K* is multiplied by a coefficient *λ* ∊ (0, 1). Then, we come back to the initial predictive time *k* and restart prediction and judgment. To avoid large tracking errors caused by too small feedback gain (this phenomenon will be analyzed in depth in Section 4), the minimum multiplier *λ*_min_ is utilized to terminate prediction. The final sub-optimal control is expressed as

(33) $$\delta (k)=\left\{\begin{array}{ccc} -\lambda^{i}K\tilde{x}(k), & \delta^{*}(k)\in [\delta_{\min},\delta_{\max}] & \\ \delta_{\min}, & \delta^{*}(k)<\delta_{\min} & \\ \delta_{\max}, & \delta^{*}(k)>\delta_{\max} & \end{array}\right.$$

where *i* is the number of times the constraints were violated;$\lambda^{i}\in [\lambda_{\min},1]$;$\delta^{*}(k)$ is the optimal control input.

### 3.3. Optimization on Preview Length Using SA Algorithm

Preview length *H* is a significant parameter in preview controller. Too long *H* may have little impact on the improvement of control performance and cause unnecessary computing burden; it can even suppress the nearest future information sharply. Meanwhile, too short *H* may incur inadequate previewed information, leading to poor tracking capability for a curvature-changing road. To obtain the optimal preview length for varying vehicle speed *v_x_* and road friction coefficient *μ*, the simulated annealing algorithm (SAA) is adopted.

Considering average lateral error and computation load, the energy function is defined as follows:

(34) $$E=\sqrt{\frac{1}{N}\sum_{i=1}^{N}e_{y}{}^{2}(i)}+\varepsilon H$$

where *e_y_* is the lateral error, *H* is the preview length representing the consumption of computation, and *ε* is a weight value. Here, we set *ε* = 0.00001.

The energy function *E* changes as the temperature *T* is reduced (this is called cooling schedule). If the temperature is not lowered slowly enough, the energy function may get trapped in a local minimum state and even not have sufficient time to reach the convergence value. To ensure the convergence and global searching ability of SAA, the annealing rate, namely temperature descending rate, is decreased exponentially.

(35) T(n+1)=wT(n)

where *T*(*n*) denotes the temperature in the *n*-*th* iteration; and *w* is the damping factor, here equaling to 0.9.

The optimization process of SAA involves four steps:

Step 1: Initialize temperature *T*, prediction length *H*, and energy function *E*. Initial *T* is set to 600. *H* is started at a random value within [1,30]; then, it is used to calculate corresponding *E*.
Step 2: Select a neighbor of current *H* randomly and compute *E*.
Step 3: If Metropolis criterion is satisfied, accept the new *H* and *E*; if not, discard them.
Step 4: Come back to step 2 if the termination condition (a minimum temperature or a maximum iteration number) is not satisfied; otherwise, the procedure terminates.

The optimization results are presented in Table 2, in which two points are noted. First, as the velocity increases, the optimal preview length becomes longer. In other words, the controller requires more future information at faster speed. Second, in low friction coefficient conditions, increasing prediction length arouses favorable performance.

## 4. Closed-Loop System Analysis

To gain insights into the proposed control algorithms, we analyze the stability, steady-state response, and frequency response in this section.

### 4.1. System Stability

The system stability is guaranteed by Lyapunov theory. Firstly, construct a Lyapunov function *L*:

(36) $$L[\tilde{x}(k)]={\tilde{x}}^{T}(k)P\tilde{x}(k)$$

where *L* satisfies the following conditions:

(37) $$\left\{\begin{array}{cc} L[\tilde{x}(k)]=0, & \tilde{x}(k)=0\\ L[\tilde{x}(k)]>0, & \tilde{x}(k)\neq 0 \end{array}\right..$$

Subsequently, calculate the increment of *L*:

(38) $$\begin{array}{cc} \Delta L[\tilde{x}(k)] & =L[\tilde{x}(k+1)]-L[\tilde{x}(k)]\\ & ={\tilde{x}}^{T}(k+1)P\tilde{x}(k+1)-{\tilde{x}}^{T}(k)P\tilde{x}(k)\\ & =-{\tilde{x}}^{T}(k)[Q+\lambda^{2i}K^{T}RK]\tilde{x}(k) \end{array}.$$

Note that *Q* is a positive semi-definite matrix, *λ* and *R* > 0; thus, Δ*L* < 0. Therefore, this closed-loop system is asymptotically stable.

### 4.2. Steady-State Response in the Time Domain

To obtain the theoretical control precision, we explore the steady-state response of the sub-optimal preview control. Substituting the control input (25) and (33) into vehicle dynamics (15) yields the closed-loop system:

(39) $$x(k+1)=(A_{k}-\lambda^{i}B_{k}K_{x})x(k)+D_{k}\rho (k)-\lambda^{i}B_{k}K_{\rho }x_{\rho }(k).$$

Z-transformation is applied to Equation (39), leading to

(40) $$X(z)={\left(zI-A_{k}+\lambda^{i}B_{k}K_{x}\right)}^{-1}[D_{k}-\lambda^{i}B_{k}K_{\rho }T(z)]P(z)$$

where *X* and Ρ are the z-transformation of *x* and *ρ*; *T*(*z*) = [1, *z*,…, *z^H^*]*^T^*. Under the assumption of constant curvature *ρ*, Ρ(*z*) in Equation (40) can be expressed as

(41) $$P(z)=\frac{z}{z-1}\rho .$$

Finally, applying the final value theorem to Equation (40) triggers the steady-state error:

(42) $$\begin{array}{cc} x_{s} & =\underset{z\to 1}{\lim}(z-1)X(z)\\ & ={\left(I-A_{k}+\lambda^{i}B_{k}K_{x}\right)}^{-1}[D_{k}-\lambda^{i}B_{k}K_{\rho }I_{(H+1)\times 1}]\rho \\ & =\left[\begin{array}{c} \frac{1}{k_{1}}(\delta_{f}+\frac{1}{\lambda^{i}}\gamma -k_{3}e_{\psi })\\ 0\\ \rho [-l_{r}+\frac{l_{f}mv_{x}{}^{2}}{2C_{\alpha r}(l_{f}+l_{r})}]\\ 0 \end{array}\right] \end{array}$$

where

(43) $$K_{x}=[k_{1},k_{2},k_{3},k_{4}],\;\delta_{f}=-B_{k}K_{\rho }I_{(H+1)\times 1\rho ,}\;\gamma =-[l_{f}+l_{r}-\frac{(C_{\alpha f}l_{f}-C_{\alpha r}l_{r})mv_{x}{}^{2}}{2C_{\alpha f}C_{\alpha r}(l_{f}+l_{r})}]\rho .$$

Equations (42) and (43) demonstrate two interesting points:

1. The sub-optimal control coefficient *λ^i^* affects the steady state *e_y_* by increasing the amplitude of *γ*. However, this situation only happens during system transient response. As the system reaches steady state, *λ^i^* approaches 1. In other words, the sub-optimal control is able to achieve steady-state error similar to the optimal control.
2. Feedback gain *K_x_* and feedforward gain *K_ρ_* can only change the steady-state *e_y_* but independent of steady-state *e_ψ_*. A higher *k*_1_ results in lowering *e_y_*. Furthermore, well-matched *k*_3_ and *K_ρ_* can lead *e_y_* to zero theoretically.

### 4.3. System Response in the Frequency Domain

To further explore the performance of sub-optimal control with constraints, the frequency response is analyzed in this section. The transfer function from curvature to path tracking errors is easily obtained based on Equation (40):

(44) $$G_{e}(z)=\frac{X(z)}{P(z)}={\left(zI-A_{k}+\lambda^{i}B_{k}K_{x}\right)}^{-1}[D_{k}-\lambda^{i}B_{k}K_{\rho }T(z)].$$

This transfer function merely determines the tracking performance, which is not enough to reflect the true response of the sub-optimal control with constraints. To look insight into the proposed control algorithm, the transfer function from curvature to constrained variables is generated.

Applying Z-transformation to Equation (31) yields

(45) $$Y_{o}(z)=(C_{ok}-\lambda^{i}B_{ok}K_{x})X(z)+[D_{ok}-\lambda^{i}B_{ok}K_{\rho }T(z)]P(z).$$

Substituting Equation (44) into Equation (45), we get the new transfer function:

(46) $$G_{o}(z)=\frac{Y_{o}(z)}{P(z)}=(C_{ok}-\lambda^{i}B_{ok}K_{x})G_{e}(z)+[D_{ok}-\lambda^{i}B_{ok}K_{\rho }T(z)].$$

**Figure 2:**
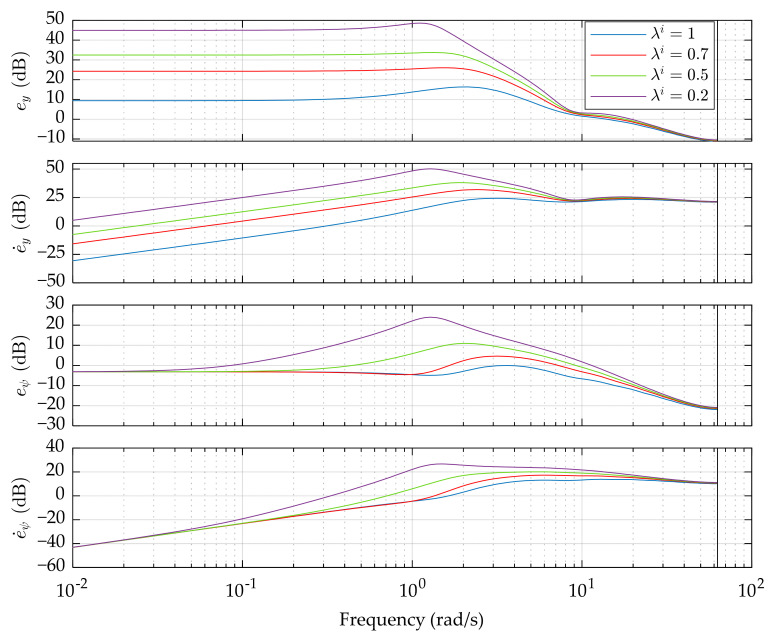
Path tracking performance in the frequency domain. Only the magnitude is presented. The vehicle speed is 20 m/s and the preview length is set to 17.

**Figure 3:**
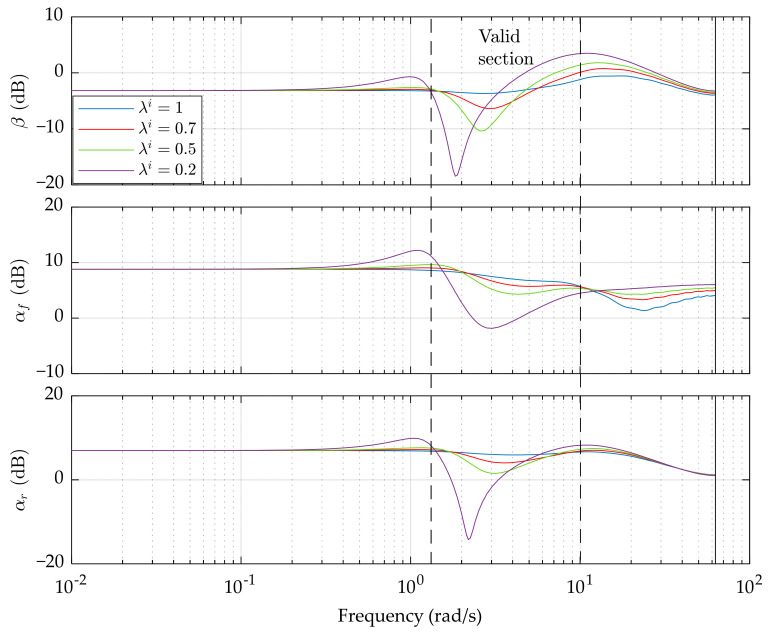
Constrained variables performance in the frequency domain. Only the magnitude is presented. The vehicle speed is 20 m/s and the preview length is set to 17.

The frequency responses of *G_e_* and *G_o_* are presented in Figure 2 and Figure 3, respectively. Based on them, two points are noted:

1. The sub-optimal control with constraints weakens the suppression of path tracking errors compared with the optimal preview control. A lower *λ^i^* usually leads to higher errors, especially at low and medium frequency. It is worth to notice that the yaw angle error *e_ψ_* is not influenced by *λ^i^* at very low frequency, which is similar to the steady-state response.
2. The proposed control has the capability of suppressing the constrained variables, resulting in the enhancement of vehicle stability. Unfortunately, this happens only in a limited frequency range, which is called the valid section. As the sub-optimal control coefficient *λ^i^* decreases, the suppression of *β*, *α_f_*, and *α_r_* becomes more obvious, but at the sacrifice of the performance beyond the valid section.

Consequently, a trade-off is determined to balance the path tracking errors and the constraints, which is to regulate the minimum of *λ^i^*. Here, we set the lower bound *λ_min_* to 0.5 for the following experiments.

## 5. Simulation and Results

### 5.1. Establishment of Simulation

To assess the proposed control algorithm, a joint simulation in CarSim–Simulink (2019–9.3) is built. The B-class hatchback vehicle model in CarSim is selected as the test object, and its basic parameters are shown in Table 3. Three types of controllers are implemented and compared in the following section. Preview-Pure is the preview controller not considering the constraints. Preview-Cons means our proposed preview controller with constraints. LTV-MPC is a linear model predictive controller, which was presented in [18]. Preview-Constraints and LTV-MPC share the same constraints, in which the range constraint of tire slip angles is [−4 deg, 4 deg] and the steering angle is limited to [−10 deg, 10 deg]. In addition, to reduce unnecessary time spent on the selections of optimal preview length under varying conditions, all controllers utilize the optimization results of Section 3.3 to predict vehicle motion. The control cycle is set to 0.05 s.

Considering performance evaluation on path tracking and handling stability, the double lane change is chosen as the reference trajectory, which represents an obstacle avoidance emergency maneuver. In this trajectory, tests under different road adhesion conditions and speeds are carried out. Specifically, the road condition with an adhesion coefficient of 0.9 is selected to represent a dry asphalt road, and the road condition with an adhesion coefficient of 0.3 is chosen to denote a snow-covered road [18,19,20,29]. Varying vehicle speeds ranging from 15 to 25 m/s are set under each road adhesion condition. The simulation results with a velocity lower than 15 m/s are not provided because in such case, little differences can be distinguished among the three controllers.

### 5.2. Results and Analysis

#### 5.2.1. Performance at Different Speeds under the High Road Adhesion Condition

This test scene aims to verify the performance of Preview-Pure, Preview-Cons, and LTV-MPC under an obstacle avoidance driving scene on a high adhesion (*μ* = 0.9, providing enough lateral force) road. A vehicle middle speed of 15 m/s, high speed of 20 m/s, and very high speed of 25 m/s are tested, respectively.

**Figure 4:**
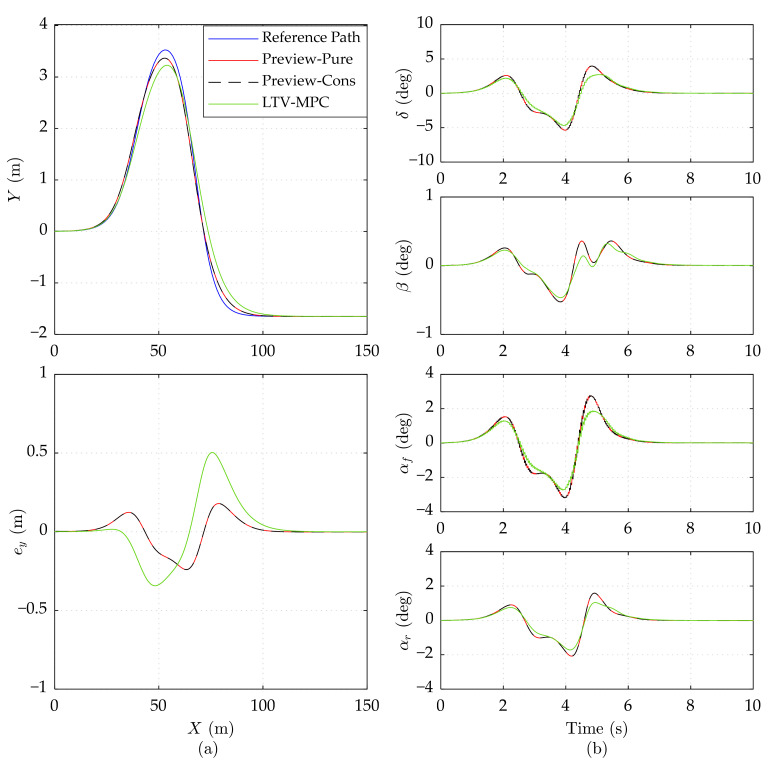
Simulation results of three controllers on the high adhesion road at 15 m/s speed. (**a**) Vehicle driving trajectory, lateral error *e_y_*. (**b**) Front wheel steering angle *δ*, mass center slip angle *β*, front wheel slip angle *α_f_*, and rear wheel slip angle *α_r_*.

As shown in Figure 4a, Preview-Pure and Preview-Cons achieve the same favorable tracking accuracy (*e_y_* no more than 0.5 m) at the 15 m/s speed, which is a bit superior to LTV-MPC. All the constrained variables are maintained far lower than the limit in Figure 4b, indicating that the constraints are not trigged. This explains why Preview-Cons is highly consistent with Preview-Pure in every facet.

**Figure 5:**
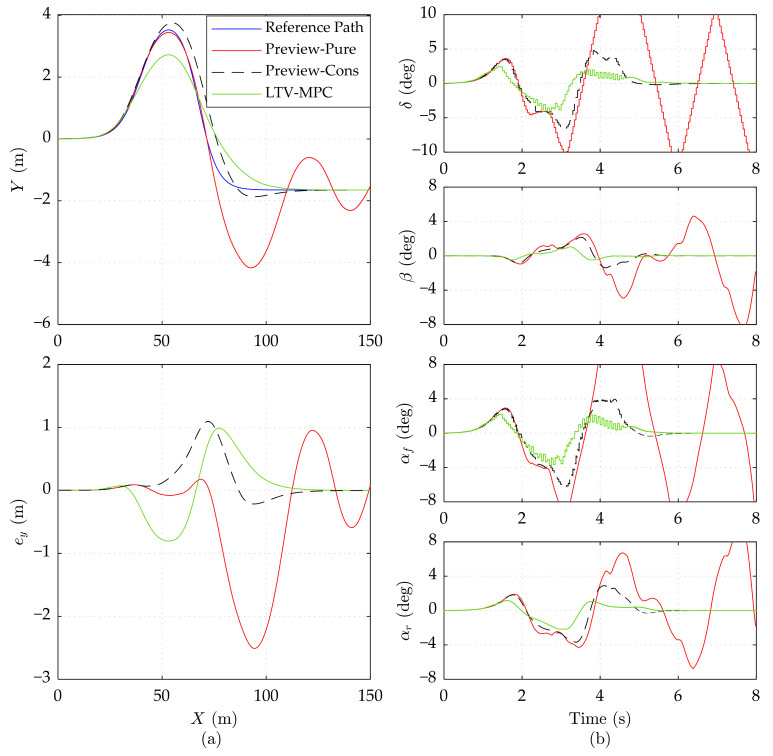
Simulation results of three controllers on the high adhesion road at 20 m/s speed. (**a**) Vehicle driving trajectory, lateral error *e_y_*. (**b**) Front wheel steering angle *δ*, mass center slip angle *β*, front wheel slip angle *α_f_*, and rear wheel slip angle *α_r_*. The results of Preview-Pure beyond range are cut off.

When the vehicle velocity comes up to 20 m/s, the superiority of Preview-Cons appears in Figure 5. We notice that before 2 s (*X* about 40 m), Preview-Pure and Preview-Cons share the same performance and control input *δ*, which implies that the constraints are not violated during this period. After 2 s, the constraints in Preview-Cons begin to intervene according to the algorithm described in Section 3.2, while Preview-Pure continues to generate the mild and aggressive turning operation (*δ* beyond 10 deg), ignoring the vehicle stability. The lack of vehicle dynamic constraints finally results in the runaway of Preview-Pure after 4 s (*X* about 80 m). Due to the suppression of constraints, the constrained variables *β*, *α_f_*, and *α_r_* in Preview-Cons are guaranteed within a stable range (between −6 deg and 4 deg), leading to pleasurable performance, as shown in Figure 5a. Note that Preview-Pure achieves better tracking accuracy before about 75 m away from the starting point, but it loses control later. In contrast, the global stability of Preview-Cons is obtained at the cost of short-term lower tracking accuracy (*X* before 75 m), which coincides with the analysis results in Section 4. As for LTV-MPC, Figure 5 manifests its conservativeness in turning operation and tracking behavior, thus giving rise to outstanding stability. However, owing to the uncertainty of numerical optimization, the control input of LTV-MPC dithers in about 3 s and 4 s.

**Figure 6:**
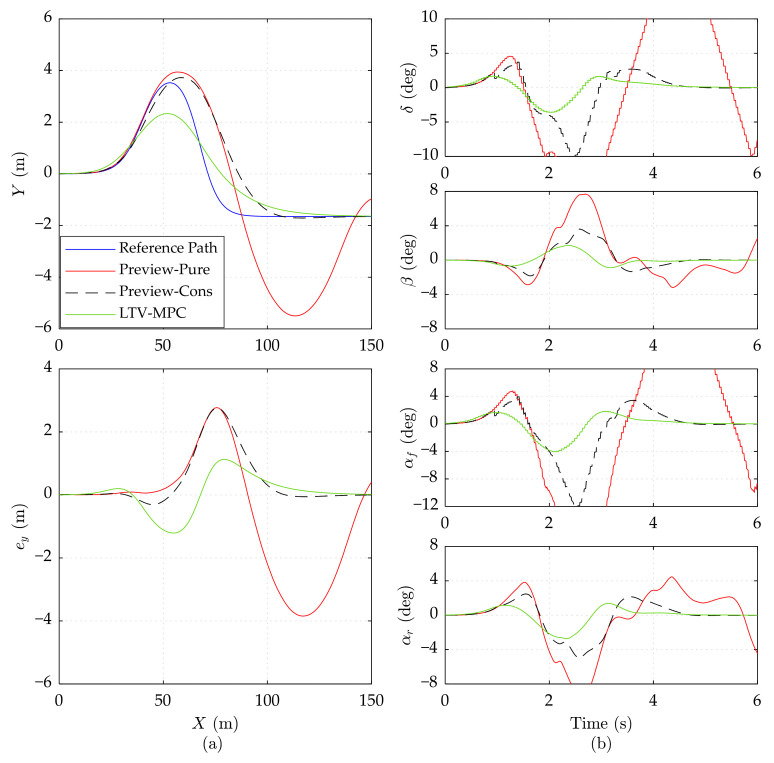
Simulation results of three controllers on the high adhesion road at 25 m/s speed. (**a**) Vehicle driving trajectory, lateral error *e_y_*. (**b**) Front wheel steering angle *δ*, mass center slip angle *β*, front wheel slip angle *α_f_*, and rear wheel slip angle *α_r_*. The results of Preview-Pure beyond range are cut off.

Figure 6 depicts simulation results on the high adhesion road at the speed of 25 m/s. We observe that Preview-Cons and LTV-MPC can still manage to track the trajectory but with a larger error in lateral position compared to corresponding results at 20 m/s. Even when the driving speed reached up to 25 m/s, the two controllers are able to stabilize the vehicle, namely, to maintain *δ*, *β*, *α_f_*, and *α_r_* in a reasonable region.

By comparing the simulation results in Figure 4, Figure 5 and Figure 6, it can be noted that LTV-MPC prefers turning a small corner to guarantee the driving stability. This action may not be suitable for an emergency obstacle avoidance. Imaging that an animal or a rock is lying in *X* = 50 m, *Y* = 2 m, the Preview-Cons can easily avoid the obstacle, but the LTV-MPC may not, especially at a speed of 25 m/s. For this reason, Preview-Con seems more competent.

In general, on a high adhesion road, Preview-Cons can achieve better tracking performance than LTV-MPC at 15 m/s and 20 m/s, and stronger driving stability than Preview-Pure at or above 20 m/s. Moreover, compared with LTV-MPC, Preview-Cons is more appropriate for an obstacle avoidance emergency maneuver.

#### 5.2.2. Performance at Different Speeds under the Low Road Adhesion Condition

**Figure 7:**
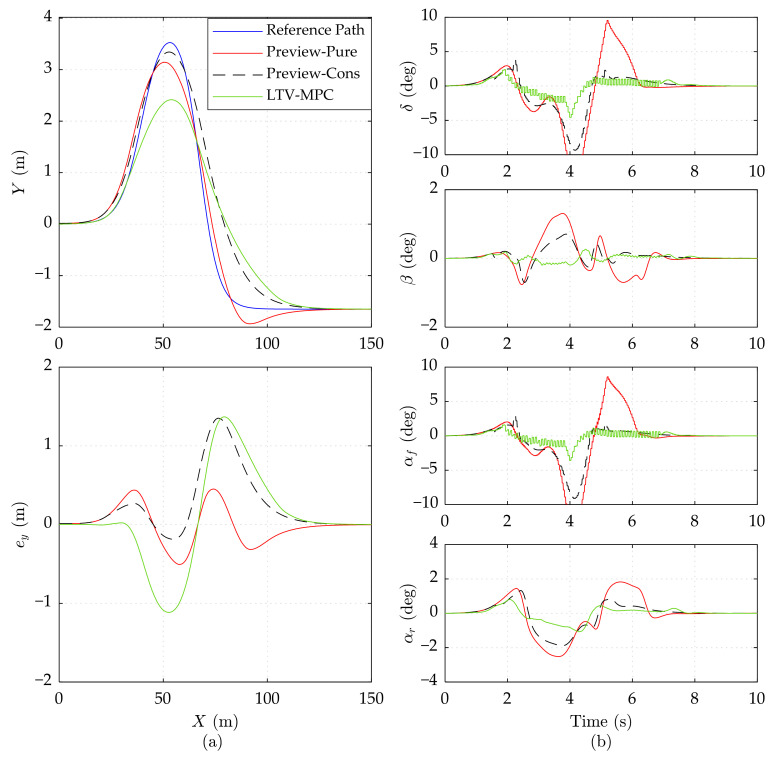
Simulation results of three controllers on the low adhesion road at 15 m/s speed. (**a**) Vehicle driving trajectory, lateral error *e_y_*. (**b**) Front wheel steering angle *δ*, mass center slip angle *β*, front wheel slip angle *α_f_*, and rear wheel slip angle *α_r_*. The results of Preview-Pure beyond range are cut off.

**Figure 8:**
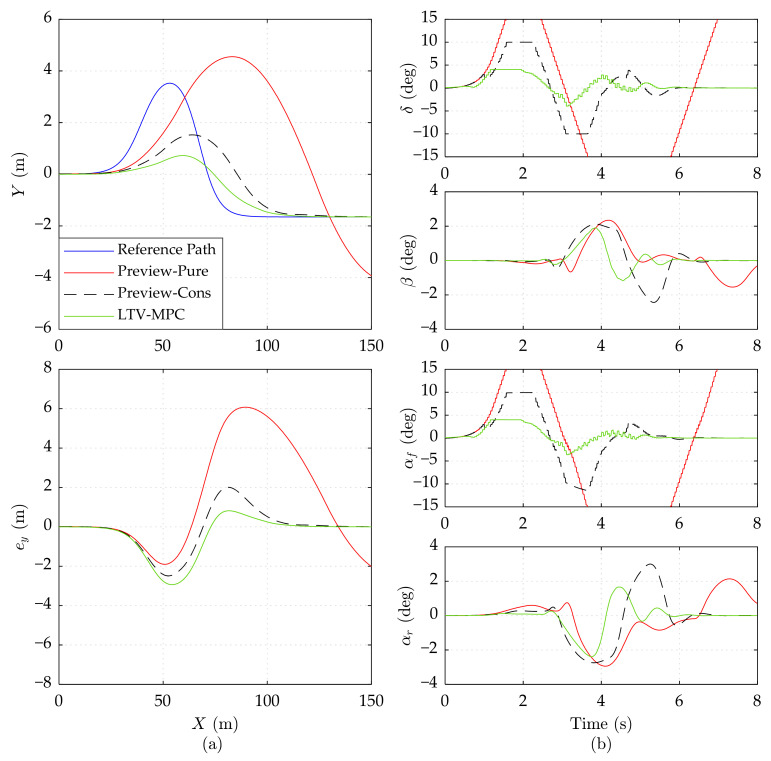
Simulation results of three controllers on the low adhesion road at 20 m/s speed. (**a**) Vehicle driving trajectory, lateral error *e_y_*. (**b**) Front wheel steering angle *δ*, mass center slip angle *β*, front wheel slip angle *α_f_*, and rear wheel slip angle *α_r_*. The results of Preview-Pure beyond range are cut off.

**Figure 9:**
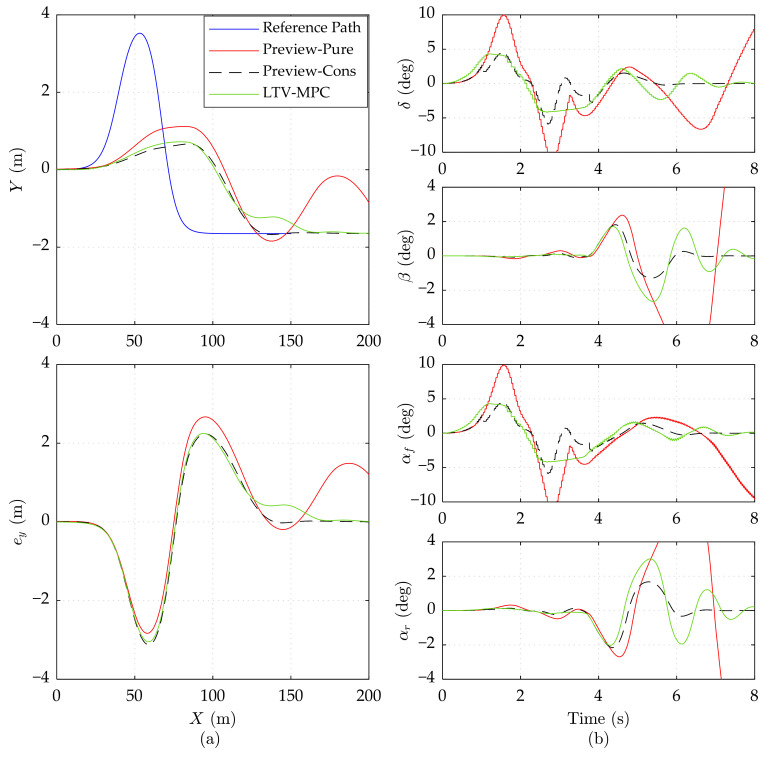
Simulation results of three controllers on the low adhesion road at 25 m/s speed. (**a**) Vehicle driving trajectory, lateral error *e_y_*. (**b**) Front wheel steering angle *δ*, mass center slip angle *β*, front wheel slip angle *α_f_*, and rear wheel slip angle *α_r_*. The results of Preview-Pure beyond range are cut off.

To further validate the effectiveness of Preview-Cons, we change the road friction coefficient *μ* to 0.3 (close to snow-covered slippery pavement). The results of the 15 m/s, 20 m/s, and 25 m/s speeds are presented in Figure 7, Figure 8 and Figure 9, respectively.

As shown in Figure 7a, the maximum *e_y_* of Preview-Cons and LTV-MPC reaches about 1.35 m, which is increased by 0.85 m, compared with Preview-Pure. Nevertheless, the control input *δ* of Preview-Pure is more aggressive than the other controllers, inducing a large slip angle *α_f_* (less than −10 deg), which is dreadful for vehicle stability. According to Figure 7b, Preview-Cons generates lowered control input *δ*, when predicting the dynamic constrained variable *α_f_* exceeding the boundary. Although this action does not ensure *α_f_* to maintain in [−4 deg, 4 deg], it sharply lowers the amplitude of *α_f_*, *α_r_*, and *β*. Unlike the dither of LTV-MPC in Figure 7b, Preview-Cons achieves smoother steering. It should be noticed that the control input *δ* of Preview-Cons suffers a slightly sudden increment in 2.2 s and 5.1 s. This action is caused by the jitter of predicted constrained variables upper and lower than the limit boundary, which does not occur frequently.

Preview-Cons and LTV-MPC manage to track the path with acceptable lateral error at high speed in Figure 8a, while Preview-Pure sideslips and departs from the reference path. Differing from other tests, high speed on a slippery road activates the hard constraint of Preview-Cons, thereby enforcing control input *δ* to keep the vehicle in a stable area.

In Figure 9, the simulation results of three controllers on the low adhesion road at 25 m/s speed are presented. It can be observed that Preview-Cons is still able to stabilize the vehicle on a low adhesion road even at 25 m/s, while Preview-Pure cannot ensure the global driving stabilization, thereby losing trajectory tracking ability after about 6 s. LTV-MPC can also guarantee the vehicle stability but with obvious oscillation in constrained variables.

Due to the limited lateral frictional force available under the low road adhesion conditions, the two preview controllers attempt increasing the control input *δ* to obtain adequate lateral force, which is a potential threat on stability. If the wheel slip angle and mass slip angle are nearby a safe region, the vehicle stability is guaranteed, as illustrated in Figure 7; otherwise, the vehicle may skid and even run away, as in the performance of Preview-Pure in Figure 8 and Figure 9. Therefore, vehicle dynamic constraints in Preview-Cons are beneficial.

In conclusion, when driving under low road adhesion conditions, the proposed Preview-Cons achieves the best overall performance compared with Preview-Pure and LTV-MPC; more specifically, it has high tracking accuracy and stability at varying speeds ranging from 15 to 25 m/s.

## 6. Conclusions

In this paper, a preview controller considering vehicle dynamic constraints is designed, analyzed, and verified. This controller regarding road curvature as preview information generates feedback control and feedforward control; thus, it is capable of responding to the upcoming bend rapidly. The constrained variables estimated and predicted from measurable error states trigger the algorithm to reduce the optimal gain. The responses in the frequency domain confirm that this operation suppresses the constrained variables, leading to stronger stability. By using the SA algorithm, the preview length is offline optimized, thereby improving the adaptability of the controller under a dynamic environment. The CarSim-Simulink joint simulations are established to verify the effectiveness of the proposed algorithm. The results show that the designed controller achieves remarkable comprehensive performance: a good level of tracking accuracy and stability under the conditions of varying velocities and road friction coefficients when compared to other controllers. As future work, we intend to consider the vehicle steering model and combine our algorithm with torque control to further enhance the driving stability.

## Figures and Tables

**Table 1:** Model parameters.

Symbol	Definition	Unit
m	Vehicle mass	kg
$I_{z}$	Yaw moment of vehicle inertia	kg·m^2^
$F_{yf}/F_{yr}$	Lateral tire force of the front/rear wheel (in vehicle body-fixed coordinate system, *oxy*)	N
$C_{\alpha_{f}}/C_{\alpha_{r}}$	Lateral stiffness of the front/rear wheel	N/rad
$\alpha_{f}/\alpha_{r}$	Slip angle of the front/rear wheel	rad
$\theta_{f}/\theta_{r}$	Velocity angle of the front/rear wheel	rad
$l_{f}/l_{r}$	Distance from center of gravity to the front/rear wheel	m
δ	Front wheel steering angle	rad
v	Vehicle speed (in inertial coordinate system, *OXY*)	m/s
$v_{x}$	Longitudinal speed (the projection of *v* in the *x* axis of *oxy*)	m/s
$e_{y}$	Lateral error from center of gravity to the reference trajectory	m
ψ	Yaw angle of vehicle	rad
$e_{\psi }$	Error of yaw angle with respect to reference path	rad

**Table 2:** Optimized preview length.

Vehicle Speed (m/s)	Road Friction Coefficient *μ*	Preview Length *H*
10	0.3	17
	0.9	4
15	0.3	28
	0.9	9
20	0.3	33
	0.9	17
25	0.3	35
	0.9	19

**Table 3:** Parameters of vehicle model.

Parameter	Value/Description
Vehicle mass	1620 kg
Front wheelbase	1.165 m
Rear wheelbase	1.535 m
Yaw inertia	3645 kg·m^2^
Powertrain	125 kW front-wheel drive
Transmission gear ratio	4.1:1
